# O antigen restricts lysogenization of non-O157 *Escherichia coli* strains by Stx-converting bacteriophage phi24B

**DOI:** 10.1038/s41598-021-82422-x

**Published:** 2021-02-04

**Authors:** A. K. Golomidova, A. D. Efimov, E. E. Kulikov, A. S. Kuznetsov, I. Sh. Belalov, A. V. Letarov

**Affiliations:** 1 Winogradsky Institute of Microbiology, RC Biotechnology RAS, Prospekt 60-letiya Oktyabrya 7 bld. 2, Moscow, Russia 117312; 2 Phystech School of Biological and Medical Physics, Moscow Institute of Physics and Technology, Moscow, Russia; 3 Faculty of Biology, Lomonosov Moscow State University, Moscow, Russia

**Keywords:** Bacteriophages, Ecological epidemiology

## Abstract

Acquisition of new prophages that are able to increase the bacterial fitness by the lysogenic conversion is believed to be an important strategy of bacterial adaptation to the changing environment. However, in contrast to the factors determining the range of bacteriophage lytic activity, little is known about the factors that define the lysogenization host range. Bacteriophage phi24B is the paradigmal model of Stx-converting phages, encoding the toxins of the Shiga-toxigenic *E. coli* (STEC). This virus has been shown to lysogenize a wide range of *E. coli* strains that is much broader than the range of the strains supporting its lytic growth. Therefore, phages produced by the STEC population colonizing the small or large intestine are potentially able to lysogenize symbiotic *E. coli* in the hindgut, and these secondary lysogens may contribute to the overall patient toxic load and to lead to the emergence of new pathogenic STEC strains. We demonstrate, however, that O antigen effectively limit the lysogenization of the wild *E. coli* strains by phi24B phage. The lysogens are formed from the spontaneous rough mutants and therefore have increased sensitivity to other bacteriophages and to the bactericidal activity of the serum if compared to their respective parental strains.

## Introduction

Temperate bacteriophages affect many aspects of the life of lysogenic bacteria through multiple mechanisms including direct or indirect influence on the host genome expression^1^, gene transduction including the recently described highly effective lateral transduction^2^ and specific mobilization of some genomic islands^3^ and other mechanisms^4^. The most known and, probably, the most ecologically significant mechanism of such influence is the lysogenic conversion^1,5^. Such a conversion occurs due to the expression in the lysogen of some prophage encoded genes, that confer the bacteria new features potentially increasing their fitness in particular habitats^5,6^. Therefore, acquisition of new prophages is believed to be one of the important strategies of bacterial adaptation in nature^4^. Bacterial antiviral systems are often expressed in phase-variable manner^7–9^. The adaptive value of such variations may be in part due to the “opening of window” for acquisition of new potentially beneficial prophages.

However, the most important factor that determines the bacteriophage host range is not the activity of the intracellular antiviral systems but the specificity of the bacteriophage adsorption. The major determinants of adsorption specificity that define bacteriophage lytic activity host range are well characterized^10^. At the same time data on the factors determining the lysogenization host range are largely missing.

The shigatoxigenic (STEC) *Escherichia coli* strains are associated with multiple foodborne diseases causing morbidity and mortality in humans^1,4,5^. STEC are zoonotic pathogens that may colonize livestock animals such as cattle without causing symptoms in them but making the agricultural environments and products dangerous for humans^10^. The majority of STEC strains belong to the O157:H7 serotype^11^, although non-O157 STEC strains have been identified and currently gain increased attention^4^ as, for example, the so-called “Big Six”—O26, O45, O1; O111,O121 and O145^12^, as well as the O104:H4 serotype that caused the well-known 2011 outbreak in Germany^13^.

STEC strains possess a number of pathogenicity factors, the foremost being the production of the Shiga toxins^11,14^ encoded by the Stx-converting prophages. Although the Stx-converting bacteriophages are quite divergent morphologically and contain genomic modules divergent by their sequences, however the genome organization of these viruses is similar to that of the bacteriophage λ, therefore the Stx phages are considered as lambdoid phages^15,16^. In these phages, the toxin gene *stx* is located downstream of the conserved gene Q encoding the antiterminator of the late gene region^15^. Toxin expression is repressed in lysogenic bacterial cells, and takes place only upon the prophage induction^15,16^. Toxin molecules lacking the signal peptide for secretion are released upon cell lysis^6,15,16^. The lysogeny in Stx-converting phages is less stable compared to the non-Stx lambdoid phages^17–20^ resulting in higher rate of spontaneous induction and in increased sensitivity to environmental factors such as DNA-damaging agents, oxidative stress or increased salt concentrations. Many antibiotics also increase the induction rate of Stx-converting prophages thus enhancing the toxin production. The increase of the toxin production may worsen the patient’s conditions and provoke the haemolytic uremic syndrome often leading to fatal outcome^1^. Therefore, the use of antibiotics to treat STEC infections remains controversial^21^.

At the same time, the STEC infections are self-limiting, and the pathogen gets spontaneously eliminated in ca. 2 weeks. The standard for the treatment of these infections relies on supportive care (symptomatic treatment, plasma exchange, infusion therapy) aiming at stabilization of the patient condition during the time required for self–curing of the infection^22^. Nevertheless in approximately 10% of the cases the hemato-uremic syndrome (HUS) is developed leading to severe kidney damage resulting in long-term morbidity or death of the patient^22^.

Thus, it is possible to speculate that the severity of the symptoms and the outcome of the disease may also depend on interaction of the Stx phage released by the STEC population with the resident *E. coli* population in the hindgut. In case of active phage multiplication in this site, the released toxin may contribute to the overall toxin load. However, Stx phages are seldom able to form plaques in vitro on isolated symbiotic gut *E. coli* strains^23^.

About 70% of Stx-converting bacteriophages are podoviruses related to the bacteriophage vb_EcoP_24B, also known as phage ϕ24B^20,24^. The phage ϕ24B lysogenization host range was reported to be much broader than its range of hosts that support plaque formation^23^. The same observations were also made for some other Stx phages^25,26^. The establishment of the lysogenic *E. coli* population in the patient’s hindgut may also represent a threat of inducible increased toxin load. The route of lateral toxin gene transmission to other (potentially) enteropathogenic *E. coli* strains adapted to gut environment may lead to emergence of new highly virulent STEC lineages^4,25^.

The secondary (terminal) receptor of bacteriophage ϕ24B has been identified as BamA protein, previously referred to as YaeT^27^, responsible for insertion of the newly synthesized beta-barrel outer membrane proteins into the bacterial outer membrane^28^. BamA protein is essential for bacterial cell viability and is therefore highly conserved. This circumstance allows speculating that a large variety of the non-Stx-producing or even non-pathogenic *E. coli* strains can be potentially lysogenized in vivo and thus get involved in STEC evolution and/or pathogenesis of the STEC-induced diseases^15^.

The available data suggest that the presence of a suitable secondary receptor is not the only factor required for successful phage adsorption and DNA delivery into the host cell. For *E. coli,* it has been shown that many O-antigen types protect the cells nearly completely against the phages not able to recognize O-antigen specifically^29–32^. This is achieved by non-specific shielding of the intimate cell surface by this structure. It was unclear how phage ϕ24B and related viruses that encode only one potential tail spike protein, gp61^20^, may penetrate the O antigen shield in diverse *E. coli* strains belonging to different O-serotypes.

Obviously, the threshold of infection efficacy required for plaque formation is much higher than for lysogenization of a small fraction of the host population. Therefore, several hypotheses can be raised to explain wide lysogenization host range of ϕ24B.

1. This phage may exploit some uncharacterized molecular mechanism to penetrate through diverse O antigens albeit with the efficiency non-sufficient for plaque formation.
2. It is also possible that the phage takes an advantage of local or temporal breaks in the O-antigen shield. The existence of a temporary phenotypical sensitivity to a virulent bacteriophage in the population of the phage resistant derivative of *E. coli* O157:H7 strain has been demonstrated previously^33^. In this system only small fraction (0.4–8%) of the cells were able to adsorb the bacteriophage but the progeny of such cells was resistant as the bulk of the population. Such phenotypical sensitivity window would be sufficient to form lysogens as it was described by James et al.^23^.
3. Alternatively, it is possible to speculate that, in the experimental conditions used by James et al.^23^, when a massive amount of the phage is added to the host cell suspension, the phage may lysogenize the small fraction of the mutant cells depleted of the O-antigen biosynthesis that is normally present in bacterial cultures^31^.

In order to discriminate between these potential mechanisms we challenged with the phage ϕ24B:cat (phage ϕ24B in which the toxin gene was disrupted by the chloramphenicol-acetyltransferase cassette) a series of environmental *E. coli* strains producing different types of O antigens. For the majority of these strains the effective non-specific protection of the cell surface by the O antigen was confirmed previously^29,30,34–39^.We compared the status of the O antigen production the lysogens formed with their parental strains.

## Results

We decided to use LPS profiling and the sensitivity to the bacteriophages with known adsorption mechanisms for evaluation of the O antigen production status of the ϕ24B lysogens generated in environmental *E. coli* isolates. To do so, liquid cultures of the O antigen producing strains 4s, HS1/2, HS3-104, F5, F17, UP1 and UP11 and of the rough strains 4sR and C600 were challenged with phage ϕ24B:cat as described by James^23^. The lysogens were then selected by plating the mixture on LB plates supplemented with 34 μg/ml of chloramphenicol. The lysogens were obtained for strains 4s, HS1/2, HS3-104, F5 and F17. No lysogens were observed on strain UP11. The lysogenization frequency was about 10^−4^ lysogen cfu/phage pfu for the rough strains and about 10^−7^–10^−6^ in O antigen-producing strains. The latter value is comparable to the frequency of spontaneous mutations by a particular gene in *E. coli* (e.g. phage-resistant mutants).

We selected 3 lysogen clones per strain and confirmed the ϕ24B prophage presence using PCR for gene 56 (the tail protein gene). For *E. coli* 4s lysogens we also performed mitomycin C induction followed by transmission electron microscopy that confirmed that a phage morphologically identical to ϕ24B was produced (Supplementary file: Fig. S1).

**Figure 1:**
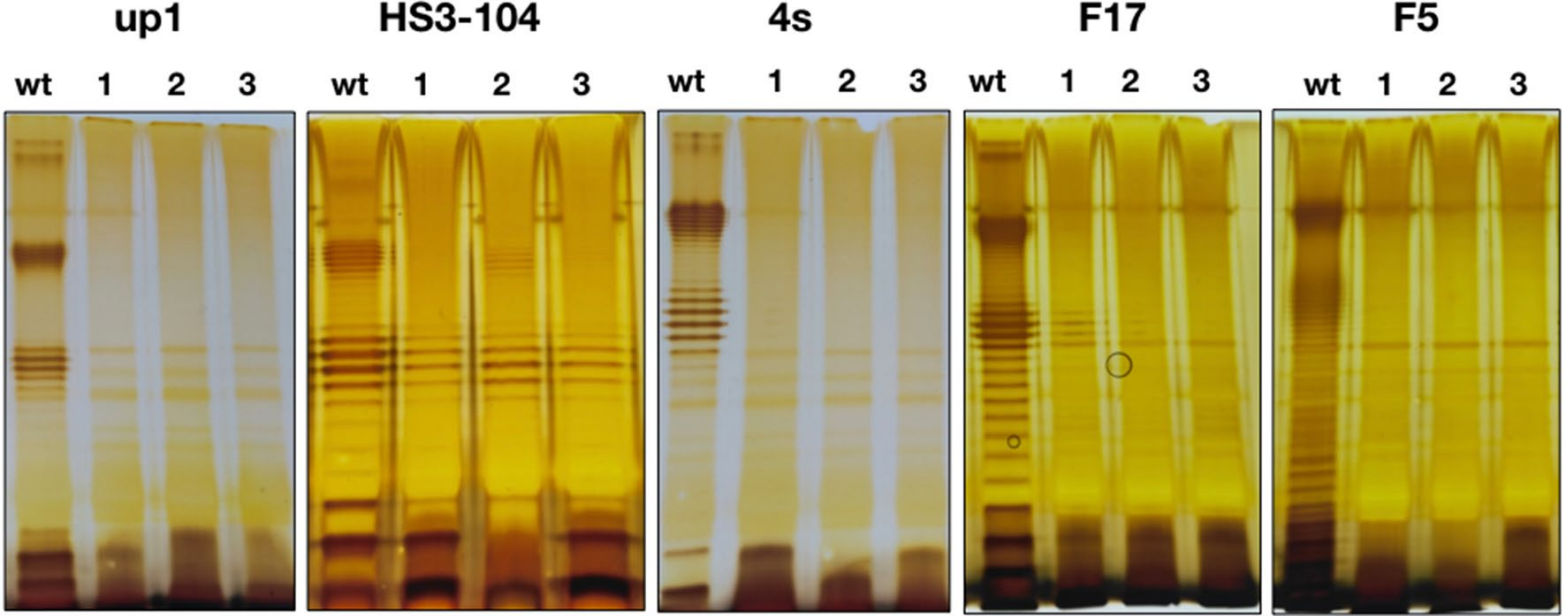
LPS profiles of the *E. coli* strains used and their derivative ϕ24B:cat lysogens. The wt lane on each of the panels—the wild type cells, lanes 1–3—three lysogenic clones for each strain.

LPS profiling of the lysogens obtained indicated that in all cases these strains did not produce O-antigen at all or the O-chain synthesis was greatly decreased compared to the parental strains (Fig. 1).

The bacteriophages that are potentially able to infect the strain but are restrained by its O-antigen can be successfully used as a probe for testing the efficacy of the O-antigen-mediated protection^31^. Previously we have developed the use of a T5-like bacteriophage DT571/2 mutant FimX lacking lateral tail fibers (LTFs) as such a probe^30^. We tested the ability of phage FimX to grow on the lawns of the lysogens obtained. This phage was not able to form plaques on the parental O-antigen—producing strains, except for F5 on which it formed plaques with an efficiency of plating (EOP) of 10^−4^ compared to the C600 strain used for FimX propagation^35^. At the same time the EOP of FimX phage on all the lysogenic cultures tested was in the range of 0.1–1.0 compared to the *E. coli* C600 strain. The gain of sensitivity to the phage FimX observed after the lysogenization was undistinguishable from other methods of rough mutant generation previously used by us in 4s or F17 strains^29,31^.

The other T5-like phages (DT57C, DT571/2, ABF and Gostya9) as well as the siphovirus 9 g demonstrated the infectivity on the lysogens derivatives of some strains that were initially resistant to these phages (Table. 1). Phage G7C that is dependent on the specific O antigen recognition for infection of *E. coli* 4s cells^30^ was not able to infect *E. coli* 4s (ϕ24B:cat) lysogenic strains in good agreement with O antigen production loss detected by the LPS profiling (Fig. 1).

**Table 1:** Sensitivity of the *E. coli* strains and their derivative ϕ24B:cat lysogens to virulent coliphages.

Phages	*E. coli* strains									
	HS3-104		4s		F5		F17		UP1	
	wt	Lysogens	wt	Lysogens	wt	Lysogens	wt	Lysogens	wt	Lysogens
DT57C	+	+	+	+	+/−	+	−	+	−	+
DT571/2	+	+	−	+	+/−	+	−	+	−	+
fimX	−	+	−	+	+/−	+	−	+	−	+
ABF	+	+	−	+	+/−	+	−	+	−	+
Gostya9	−	+	−	+	+	+	−	+	−	−
G7C	−	−	+	−	−	−	−	−	−	−
T5	−	+	−	+	−	−	−	+	−	+
9 g	−	+	−	+	+	+	+	+	−	+

“+”—the phage plaques are formed at the EOP > 0.1 in respect to plating on the optimal host strain, “−”—no plaque formation observed, “+/−”—very small turbid plaques with the EOP < 10^−3^.

We concluded that the lysogenization of the wild O-antigen-producing strains of *E. coli* is associated with the loss or reduced amount of the O antigen. The most probable explanation of this effect is that the phage infects and lysogenizes naturally occurring rough mutants that are normally present in the *E. coli* cultures (see refs 29 and 31 and the literature cited therein). However, an alternative explanation that the prophage acquisition leads to severe impairment of the O antigen production remains possible.

**Figure 2:**
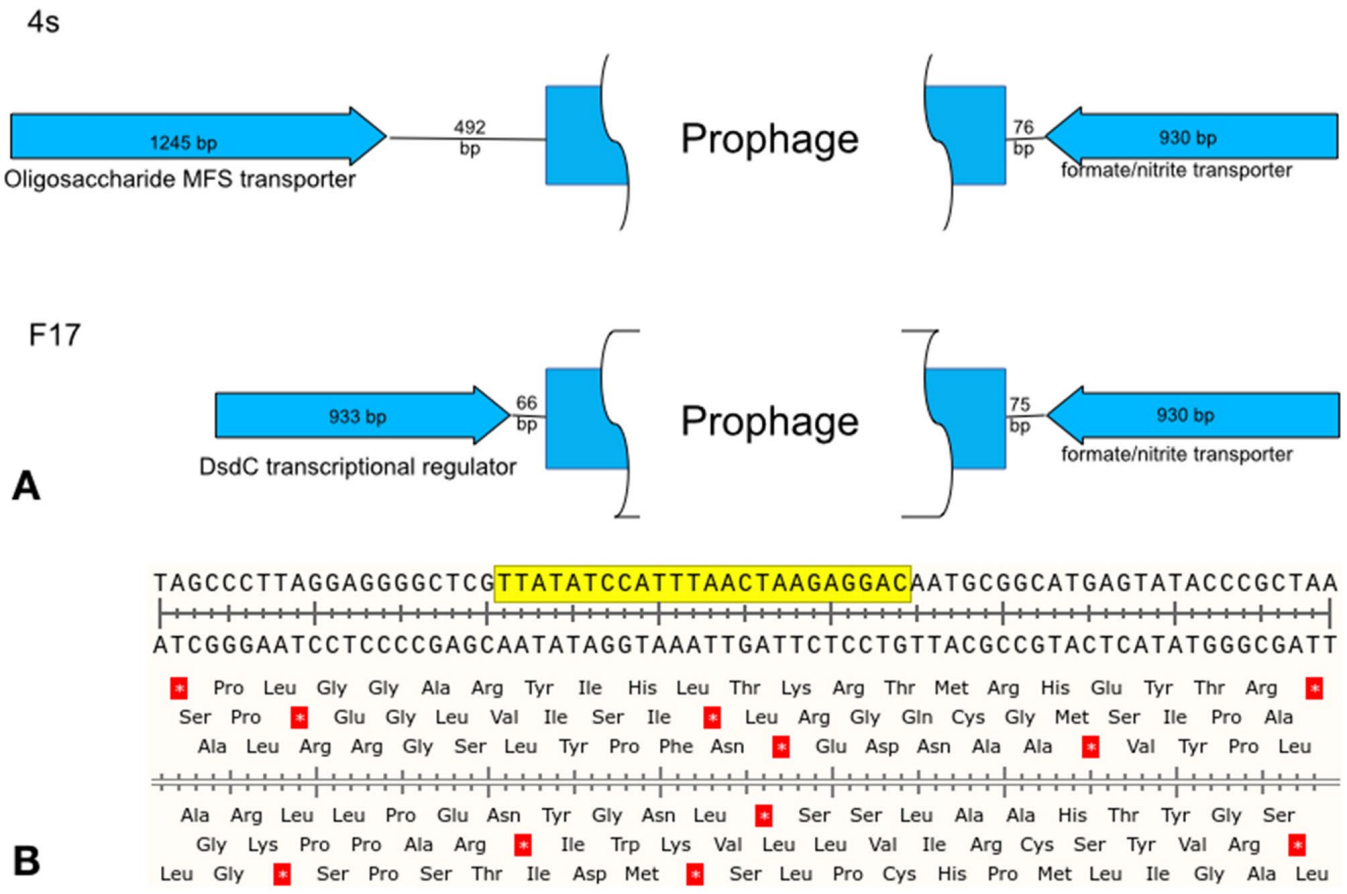
(**A**) The genetic organization of the chromosomal locus of phi24B prophage integration in the *E. coli* strains 4s and F17. (**B**) The nucleotide sequence of the conserved integration site. The yellow box—the sequence that is duplicated during the integration to flank the prophage in the lysogens.

To further address this question, we extracted the DNA from the lysogens obtained on the strains 4s and F17 and submitted it for the whole genome using Oxforde Nanopore MiniIon sequencer. The contigs were assembled and the prophage integration sites were determined. In both strains the prophage was integrated by a conserved *attB* sequence located in the spacer between two oppositely directed genes (Fig. 2). These genes are not involved into the O antigen biosynthesis pathway and are located at long distances (345 kbp and 336 kbp in 4s and F17 strains respectively) from the O antigen clusters previously described for these strains^29,36^. Thus the prophage integration itself does not directly break the genes or transcription units responsible for the O antigen synthesis.

**Figure 3:**
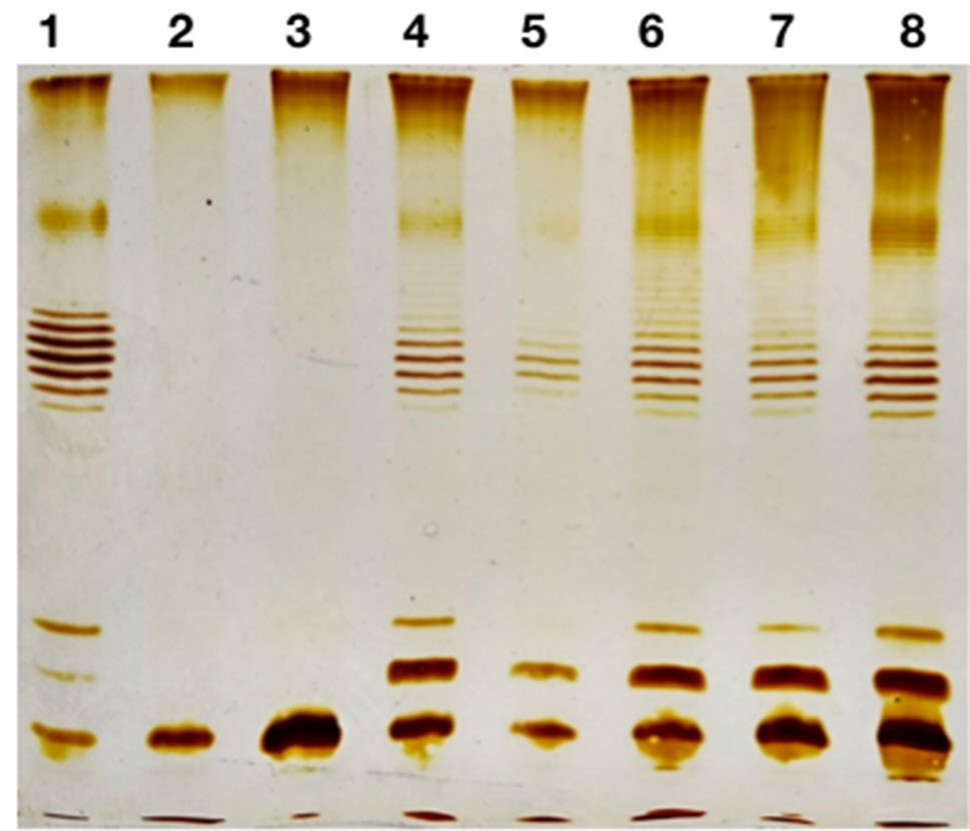
Complementation of the *wclH* mutation from the plasmid on phi24B lysogens background. Lanes 1—*E. coli* 4s wild type, 2—4sR, 3—4sR (phi24B), 4—4sR:pWclH, 5—the same as lane 4 with less sample load, 6—8 three clones of 4sR(phi24B):pWclH.

However, it is possible that the prophage interferes with this process at the metabolic level. To test such possibility we used the complementation system previously developed by us in *E. coli* 4s strain. In this strain a rough mutant, refered to as 4sR, was described. The rough phenotype of the 4sR strain was due to the gene *wclH* inactivation by the insertion of an IS1-like mobile element, and the complementation by the expression of *wclH* gene from the plasmid was shown to restore O-antigen producing phenotype^29^. We lysogenized *E. coli* 4sR with the phage ϕ24B and transformed the lysogen by the pWclH plasmid. The O antigen production status was then assessed by LPS profiling (Fig. 3). The LPS profile, similar to the wild type E. coli 4s strain was restored both in the parental 4sR strain and in all three lysogenic clones tested. We concluded that the ϕ24B prophage does not significantly interfere with the O antigen biosynthesis. Thus, the rough phenotype is not induced by the prophage acquisition but but is selected by the lysogenization procedure.

**Figure 4:**
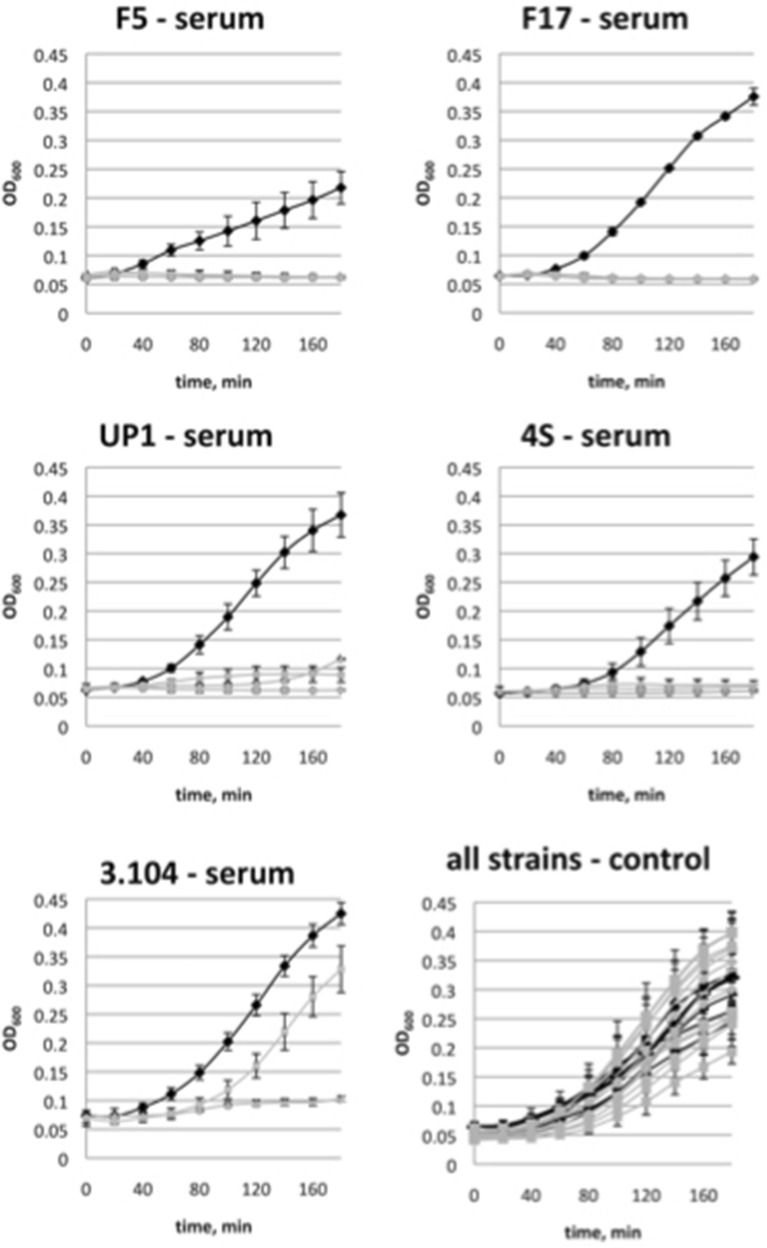
Sensitivity of the *E. coli* strains and their derivative ϕ24B:cat lysogens’ growth to the horse serum bactericidal activity. Black lines—the wild type strain, grey lines—three lysogenic clones tested for each original strain.

Since the O antigen synthesis compromised strains are believed to be more vulnerable to immunity factors, we decided to measure the susceptibility of the lysogens obtained to the bactericidal activity of the horse serum (SBA). All the wild type strains were resistant to SBA in our conditions (Fig. 4). Their cultures grew in presence of the serum as well or even slightly more rapidly than in the control experiment. In the absence of the serum the lysogenic strains showed the growth rates close to their cognate wild type strains. At the same time the growth of the lysogens was almost completely abolished in the presence of the serum (Fig. 4).

Only one of the lysogenic clones tested, the derivative of the strain HS3-104, was able to grow significantly in presence of the horse serum, though the rise of the optical density was delayed and the growth rate was significantly lower than in the parental strain (Fig. 2). This result can be explained by the fact that in HS3-104 lysogens the O antigen synthesis was strongly decreased but not completely abolished (Fig. 1). So, the actual synthesis of O-polysaccharide could be upregulated in this particular clone in the conditions of the experiment of SBA sensitivity measurement.

## Discussion

The results obtained allow us to conclude that lysogenization by the phage ϕ24B of diverse *E. coli* strains producing O antigens was not due to an unusual ability of this virus to penetrate the O antigen shield, but was mediated by spontaneous formation of bacterial rough mutants or of mutants with significantly compromised O antigen biosynthesis. It is not clear why the lysogenization was not effective for some strains. The activity of antiviral systems, such as restriction-modification, avoiding the lysogenization at stages after the viral DNA penetration into the cell^46^, cannot be excluded. Also, the effect may be due to point mutations present in BamA protein or lower frequency of rough mutants in particular strains.

In the conditions of our experiment, the high concentration of bacteriophage used allowed almost all the cells potentially susceptible to the phage to be infected. However, in vivo the populations of *E. coli* are very unlikely to face such a massive viral attack. The fraction of rough mutants in natural habitats is hard to estimate, but we can speculate that it should be lower than in in vitro conditions because such mutants have compromised protection not only from the phage attack but also from immune system agents such as serum bactericidal activity^47–49^ and from other environmental factors^32^ and therefore should be counter-selected. Moreover, if Stx-phage lysogens were formed by infection of such rough mutants, their expected fitness and/or virulence would be significantly lower than that of the parental strains. These strains, noteworthily, were highly sensitive to SBA of the horse serum to which the parental O-antigen producing strains were completely resistant. Therefore, the lysogens for the ϕ24B phage are expected to have reduced virulence. Thus, the factor of non-specific protection of the bacterial cells by the O antigen should not be neglected during the evaluation of the potential significance of Stx-converting phage transmission in nature (as it currently is neglected in many studies^25,26^).

We also should note that the lysogenization by ϕ24B:cat appears to be a simple and efficient procedure for selection for mutants with compromised or completely abolished O antigen synthesis. This procedure may be particularly valuable for the researchers working with field isolates of *E. coli* for which the genomic sequences are not yet available and/or in which other rapid techniques such as recombination with PCR fragments for genes knockout^50^ are frequently less effective than in laboratory *E. coli.*

## Methods

### E. coli and bacteriophage strains and their cultivation

The *E. coli* strain MG1655 lysogenized for phage ϕ24B:cat was a kind gift of Prof. G. Wegrzyn, University of Gdansk, Poland. Phage T5 was a gift of Dr. V. Ksenzenko (Institute of protein research RAS, Puschino-na-Oke, Russia). We previously described T5-like bacteriophages of DT57C species and their LTF mutants^30^. These include: phage DT57C, phage DT571/2, DT571/2 ltfA^−^ mutant lacking the LTFs (hereafter FimX) and DT571/2 mutant ABF that carries LTF non-branched LTF with only one receptor-binding domain (instead of two such domains on the branched LTFs of the phages DT57C or DT571/2). Bacteriophage 9 g, a siphovirus representing the type strain of the genus Nonagvirus^40^. Gostya9 is a T5-like bacteriophage that was shown to recognize a different secondary receptor distinct from the receptors of the phages T5, DT57C and 9 g^41^. Bacteriophage G7C, a N4-related podovirus specifically recognizing O antigen of *E. coli* 4s strain was isolated and characterized by us previously^42,43^. We isolated all the above-mentioned phages except for T5 and engineered phage mutants from horse feces as it described in the corresponding publications cited above.

The wild *E. coli* strains were previously isolated by us from horse feces and characterized. These were 4s (O22)^29^, HS1/2 (O87)^37,44^, HS3-104 (O81)^39^, F5 (O28 ab)^35^, and F17 (new O-serotype)^36^. The clinical uropathogenic *E. coli* isolates UP1 and UP11 were received from the clinical microbiological facility of the Institute of Epidemiology (Moscow, Russia). UP11 strain was further identified as an O5 O-antigen producer^34^.

The ability of the strains to produce O antigens was controlled by LPS profiling as described in Kulikov et al. (2019)^31^.

*E. coli* 4s and F17 rough variants 4sR (a *wclH* mutant of 4s)^29^ and F17 *wbbL*^−^^36^ were engineered by us previously.

All the *E. coli* strains were cultured on LB medium (trypton 10 g, yeast extract 5 g, NaCl—10 g, distilled H_2_O—up to 1 l). This medium was supplemented with 15 g of bacto-agar per 1 l for plates or with 6 g of bacto-agar for top agar.

Bacteriophage FimX was propagated on *E. coli* 4sR and enumerated using the conventional double-layer plating technique.

Bacteriophage ϕ24B:cat was obtained by mitomycin C induction of *E. coli* MG1655 (ϕ24B:cat) strain. For this procedure, the overnight culture of the lysogen was grown in the presence of 34 μg/ml of chloramphenicol. Then 300 ml of LB in 500 ml Erlenmeyer flask was inoculated with 3 ml of the overnight culture (N.B.—this volume ratio gave a better phage yield than conventional conditions with better aeration). The culture was grown in the orbital shaker at 220 rpm, 37 °C up to OD_600_ = 0.2. The mitomycin C was then added up to 1 μg/mL and the incubation was continued overnight at the same conditions. After the incubation, lysis of the culture was observed. The lysate was cleared by centrifugation at 15,000 × g for 15 min. The supernatant was collected, PEG-precipitated^45^, and resuspended in 3 mL of SM buffer (Tris–HCl pH 7.5–10 mM, NaCl—50 mM, MgCl_2_—10 mM, gelatin—5 g/l). The phage stock was titered and used in these experiments.

For titration of the phage ϕ24B:cat, a modified double-layer technique was used. The top-layer medium contained 4 g/l of the bacto-agar (instead of 6 g/l) and was supplemented with CaCl_2_ up to 5 mM. The bottom layer was supplemented with 2.5 μg/ ml of chloramphenicol. 300 μg of log-phase culture of *E. coli* C600 (OD_600_ = 0.6) was used for the lawn inoculation.

### Lysogenization of the *E. coli* strains

This procedure was performed as described in James et al.^23^ with minor modifications. Briefly, a mid-log liquid culture of an appropriate strain was grown in LB medium, the phage was added at a multiplicity of 5 pfu/host cfu, and the mixture was incubated at 37 °C for 30 min. After the incubation, the cells were spun down in a table-top centrifuge (10,000×*g*, 1 min), the cells were resuspended in LB, washed twice with LB to remove non-bound phage and plated on plates supplemented with 34 μg/ml of chloramphenicol for lysogen selection.

#### Sequencing of the bacterial genomic DNA

DNA was extracted using the Wizard DNA extraction kit (Promega Corporation, USA). The sequencing was performed with MinION sequencing (Oxford Nanopore Technologies, UK). The sequencing libraries were prepared using the ligation sequencing kit (catalog number SQK-LSK109) and native barcoding expansion kit (catalog number EXP-NBD114) and run in a FLO-MIN106 flow cell. Reads were base called trimmed and demultiplexed using Guppy v. 3.2.5. The contigs were assembled with Flye 2.5.

### LPS profiling

By SDS-PAGE electrophoresis was performed as recently described^31^. The lysogens cultures were grown for this prodecure on the LB medium without antibiotic to avoid possible chloramphenicol-induced alterations of the LPS synthesis.

### Serum bactericidal activity (SBA)

Against different strains was measured as follows. The blood samples obtained from clinically healthy horses were used. These samples were collected by a qualified veterinary doctor for the the animal healthcare purposes unrelated to our study, and we used the excess of the serum in our experiments. According to the local legislation this is not considered as the experiment on animals and does not require specific ethical approval. Therefore, we formally confirm that all invasive or non-invasive experimental protocols involving experimental animals were carried out with strict adherence to Russian legislation in this area, and in complete accordance with the current regulation status. The samples were collected into the yellow-cap vacuum tubes with the clot activator (Elamed, Moscow, Russia). The serum was separated by centrifugation at 1600×*g* for 10 min. The serum was stored at + 4 °C and used for the tests within 24 h. For the SBA assessment, the wells of 96-well plate containing 175 μl of LB medium and 25 μl of the serum were inoculated with 5 μl of the corresponding strain mid-log phase culture (OD_600_ = 0.6) and the plates were incubated at 37 °C in an automated plate reader with agitation. The OD_600_ was recorded every 30 min. In the control experiment the same volume of physiological saline replaced the serum. The whole experiment was triplicated.

## Supplementary Information

Supplementary Information
