# Supplementary material for: O antigen restricts lysogenization of non-O157 Escherichia coli strains by Stx-converting bacteriophage phi24B
Source: Sci Rep. 2021 Feb 4;11:3035. doi: 10.1038/s41598-021-82422-x (PMC7862636; doi:10.1038/s41598-021-82422-x)
Supplement: Supplementary file 1 — Supplementary Information [file 41598_2021_82422_MOESM1_ESM.pdf]

O antigen restricts the lysogenization of the non-O157 *Escherichia coli* strains by Stx-converting bacteriophage phi24B

Golomidova A.K.[a], Efimov A.D.[a], Kulikov E.E.[a,b], Kuznetsov A.S.[a,c] , Belalov I.Sh.[a], Letarov A.V.[a,c*]

[a]*Winogradsky Institute of Microbiology RC Biotechnology RAS, Moscow, Russia*

[b]*Phystech School of Biological and Medical Physics, Moscow Institute of Physics and Technology, Moscow, Russia*

[c]*Faculty of Biology, Lomonosov Moscow State University, Moscow, Russia*

[*]*Prospekt 60-letiya Oktyabrya 7 bld. 2., 117312, Moscow, Russia. letarov@gmail.com*

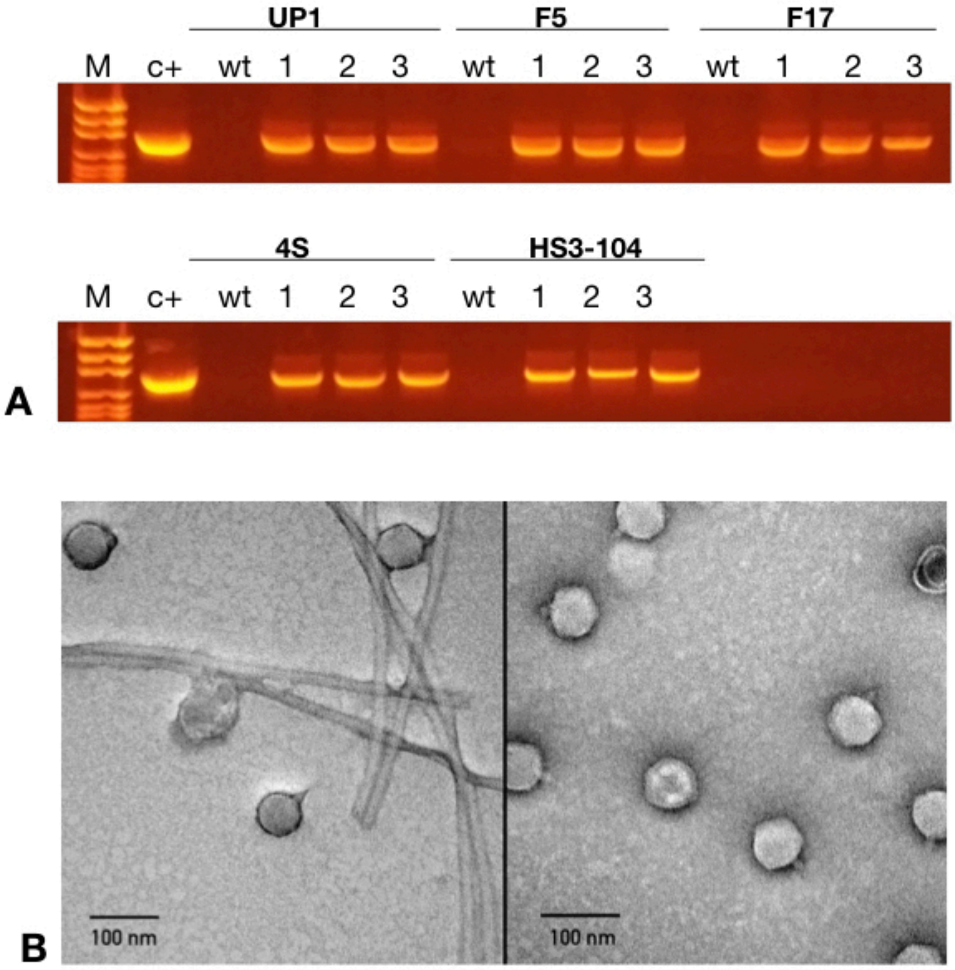

Supplementary Figure 1. A. Testing of the host strains and their lysogenic derivatives by the PCR for phi24B g56 sequence. M – marker, c+ – phage 24B, wt – the non-lysogenic culture of the corresponding strain, 1-3 three lysogenic clones of the corresponding strain. B. Morphology of the phage particles obtained by the induction of *E. coli* K12 lysogen (left) and *E. coli* 4s lysogen (right).

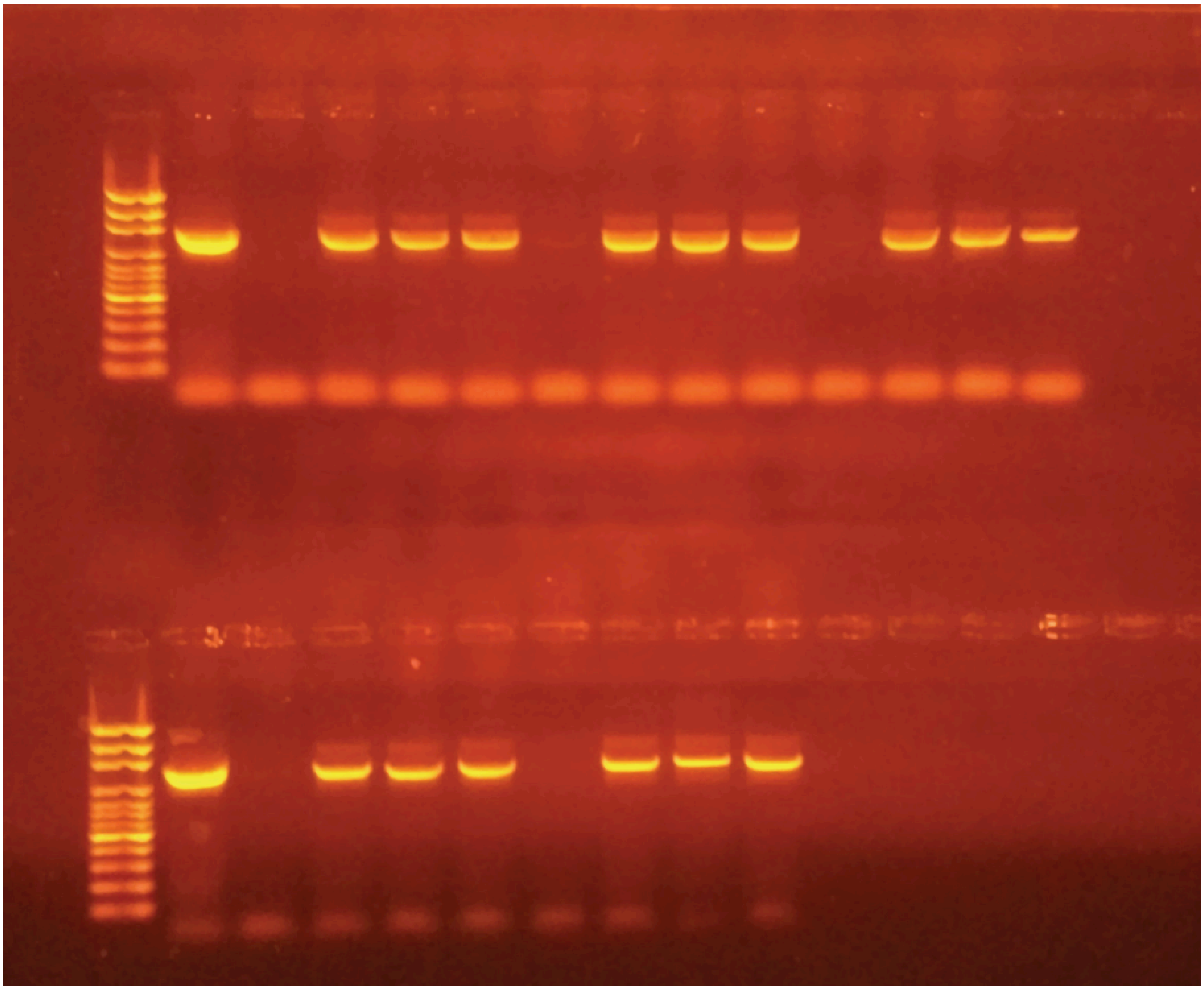

Gel image, used for creation of the **Supplementary Figure 1**

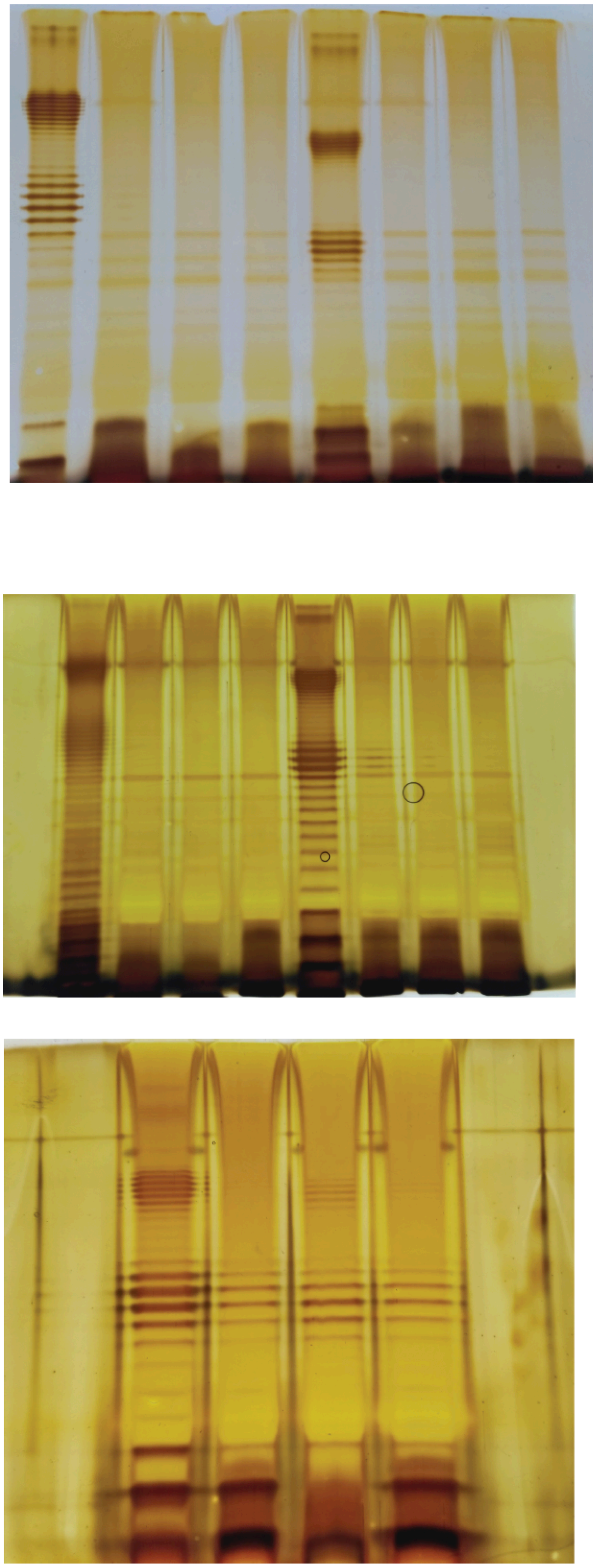

Original gel images, used for cteartion of the **Figure 1**.
